# Gastrointestinal Perforation After Solid Organ Transplantation: A Case Series

**DOI:** 10.7759/cureus.59977

**Published:** 2024-05-09

**Authors:** Duong Duc Hung, Le Nguyen Vu, Nguyen Viet Hoa, Ninh Viet Khai, Nguyen Quang Nghia, Tran Que Son, Nguyen Kim Dan

**Affiliations:** 1 Cardiovascular Surgery Center, Viet Duc University Hospital, Ha Noi, VNM; 2 Surgery, University of Medicine and Pharmacy - Vietnam National University, Ha Noi, VNM; 3 Organ Transplantation Center, Viet Duc University Hospital, Ha Noi, VNM; 4 University of Medicine and Pharmacy, Vietnam National University, Ha Noi, VNM; 5 Pediatric Surgery Department, Viet Duc University Hospital, Ha Noi, VNM; 6 Surgery Department, Hanoi Medical University, Ha Noi, VNM; 7 Digestive Center, Bach Mai Hospital, Ha Noi, VNM

**Keywords:** case series, acute peritonitis, solid organ transplant, intestinal perforation, gastrointestinal complications

## Abstract

Although organ transplantation is associated with significant survival rates and cost benefits, postoperative complications still occur. Gastrointestinal complications, including those involving the stomach and intestines, account for 1-6% of posttransplant complications, with intestinal perforation specifically accounting for approximately 9%, depending on the center. In Vietnam, there are no comprehensive reports on these complications. Therefore, we report three clinical cases of gastrointestinal perforation following transplantation.

Three cases of intestinal perforation are described in this case series. In 2023, a 16-year-old female patient who underwent heart transplantation for congenital heart disease was diagnosed with intestinal perforation on the 12th day. The patient required continued blood filtration support after surgery. In 2018, six days after liver transplantation, a 56-year-old male patient was diagnosed with intestinal perforation, which was subsequently repaired, and the ends of his intestines were removed. The patient was discharged in stable condition after 30 days. In 2017, five days after kidney transplantation, a 46-year-old female patient was diagnosed with intestinal perforation, which was repaired, and the perforation site was left open. The patient was discharged in stable condition after 40 days.

Intestinal perforation is a relatively rare, but not uncommon, complication. Early diagnosis is challenging due to nonspecific clinical symptoms and signs. Considering the possibility of intestinal perforation and obtaining early abdominal computed tomography imaging can help prevent delayed diagnosis.

## Introduction

Organ transplantation is an alternative treatment method for patients with end-stage organ failure. According to statistics, the number of solid organ transplants in Vietnam is growing [1]. Although organ transplants offer high survival rates and financial advantages, postoperative complications are inevitable. Gastrointestinal complications account for 1-6% of postoperative adverse events, with gastrointestinal perforation accounting for 9% of such cases, depending on the organ. Locations of gastrointestinal perforation include the colon, small intestine, stomach, and duodenum [2]. We found no previous reports of this complication in Vietnam. At our hospital, three clinical cases of gastrointestinal perforation after transplantation have been reported. This article aims to share the diagnosis and treatments of these clinical cases to contribute more experience in the early diagnosis of this disease.

## Case presentation

Case 1: ileal perforation after heart transplantation

**Figure 1:**
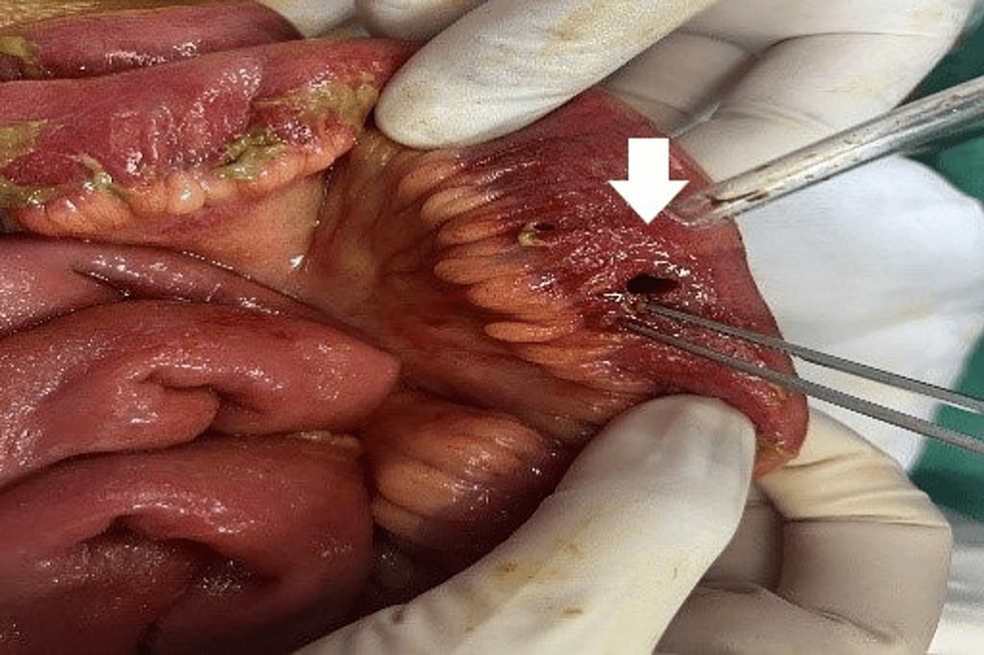
Perforation in the free area of ​​the jejunal loop (white arrow).

The patient was a 14-year-old female from Lai Chau, a province in the North of Vietnam, with a height of 155 cm, weight of 65 kg, and body mass index (BMI) of 27 kg/m^2^. Her medical history included complicated congenital heart disease, and she had undergone two procedures (the Glenn procedure at 17 months of age and the Fontan procedure at five years of age). For the past year, the patient had felt tired and experienced dyspnea (New York Heart Association (NYHA) IV), edema, and oliguria. Doppler echocardiography revealed an ejection fraction of 28%, and there was no response to drug treatment for a few months. Consequently, the patient was diagnosed with end-stage heart failure and was scheduled for heart transplantation. Subclinical results before surgery revealed the following high-risk factors: fibrosis stage, F4 cirrhosis; positivity for the virus Epstein-Barr virus (EBV) IgG and cytomegalovirus (CMV) IgG; and positivity for the hepatitis C virus (HCV). HCV treatment for three months according to the pretransplant regimen with sofosbuvir/velpatasvir at a dose of one tablet/day for the first three months, followed by viral load measurement before transplantation if below threshold or negative, could not be measured. Every month for the first three months, the patient’s viral load was measured and her liver function was monitored. The patient received a heart transplant from a brain-dead donor on February 26, 2023. The donor was a 35-year-old male with the same blood type (height: 170 cm, weight: 66 kg, BMI: 29.7 kg/m^2^). The patient’s condition was severe, and there were numerous risk factors such as NYHA IV heart failure, fibrosis stage, F4 cirrhosis; positivity for the virus Epstein‒Barr virus (EBV) IgG and cytomegalovirus (CMV) IgG; and positivity for HCV. The transplant technique was very complicated because of the need for a heart transplant on a congenitally diseased heart, the myocardial ischemia time of more than eight hours, and the requirement for a heart-lung machine. During and after surgery, the hemodynamics were unstable, and the patient received high doses of cardiac support and vasopressors. After surgery, there was a multiorgan failure (heart, liver, kidney, and lungs). The immunosuppressive drugs used included steroids, calcineurin inhibitors, and mycophenolate mofetil (MMF). The patient received intensive resuscitation treatment, mechanical ventilation, and intravenous nutrition. After seven days of mechanical ventilation, the patient’s condition stabilized and improved. However, on the eighth day, she had black stools and was treated with an intravenous infusion of Nexium (five vials/day for three consecutive days). A blood test revealed a hemoglobin level of 111 g/L and a platelet count of 70 T/L. On the 10th day, the patient was extubated, and no black stools were noted. On the 12th day, the patient experienced abdominal pain, black stools reappeared at 11 pm on the same day, and gastroscopy revealed superficial gastric ulcers without bleeding. Blood tests revealed a white blood cell count of 21 g/L, hemoglobin of 97 g/L, platelets of 89 T/L, and Co of 16 ng/mL. On the 14th day, the patient experienced less abdominal pain and reduced black stools. Subsequent blood tests showed a slight increase in serum amylase, but clinical suspicion for acute pancreatitis was low. As the abdominal pain persisted, the patient underwent hemodialysis due to multiorgan failure. A computed tomography (CT) scan of the abdomen revealed intra-abdominal gas, suggestive of visceral perforation. Emergency surgery revealed three perforations in the small intestine, 2.5 m from the angle of Treitz, with significant amounts of digestive fluid (Figure 1).

**Figure 2:**
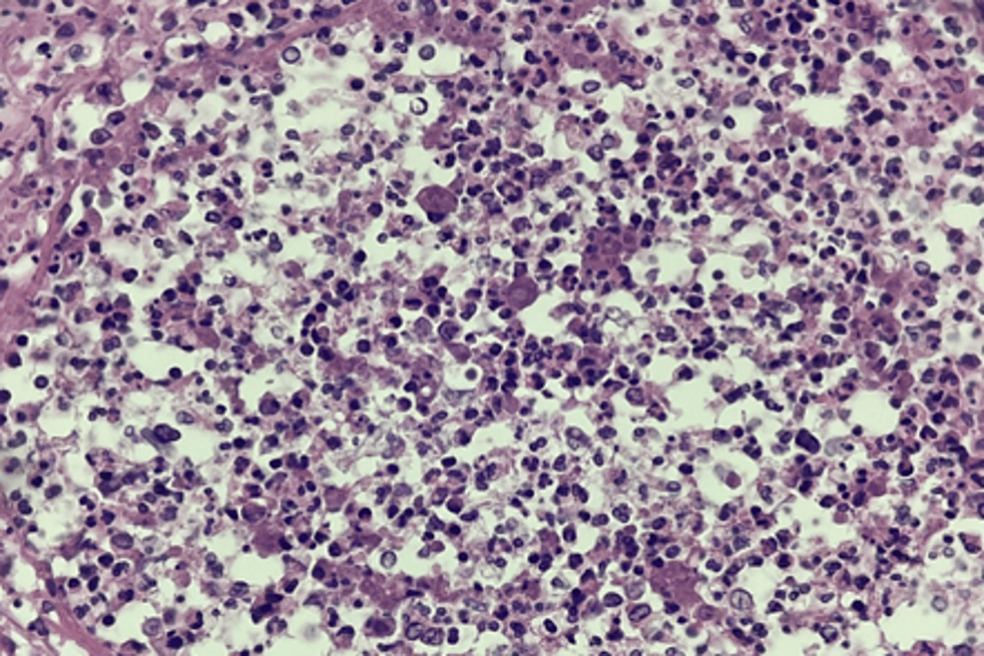
Histopathological examination showing fungal hyphae on intestinal necrosis.

The liver showed evidence of fibrosis in two lobes. Following bowel resection surgery, the patient regained consciousness after three days, with stable vital signs and a return to black stools. Drainage from the abdomen did not reveal any signs of digestive fluid. A follow-up CT scan showed no evidence of active bleeding in the abdomen, and a colonoscopy revealed multiple inflamed patches in the colon without ulcers or bleeding. Blood culture revealed *Trichophyton* species and *Klebsiella pneumoniae*. Intraoperative fluid culture revealed *Klebsiella pneumoniae* and *Acinetobacter baumannii*, with histopathological examination showing fungal hyphae on intestinal necrosis (Figure 2). *Candida stellatoidea* was isolated, for which fluconazole 200 mg per day was administered intravenously.

The patient continued to receive antibiotic therapy, parenteral nutrition, hemodialysis, and immunosuppressive drugs as per the protocol. The abdominal drains were removed, and the patient was managed with oral and enteral nutrition, with stable vital signs and a soft abdomen without further black stools.

Case 2: sigmoid colon perforation after liver transplantation

**Figure 3:**
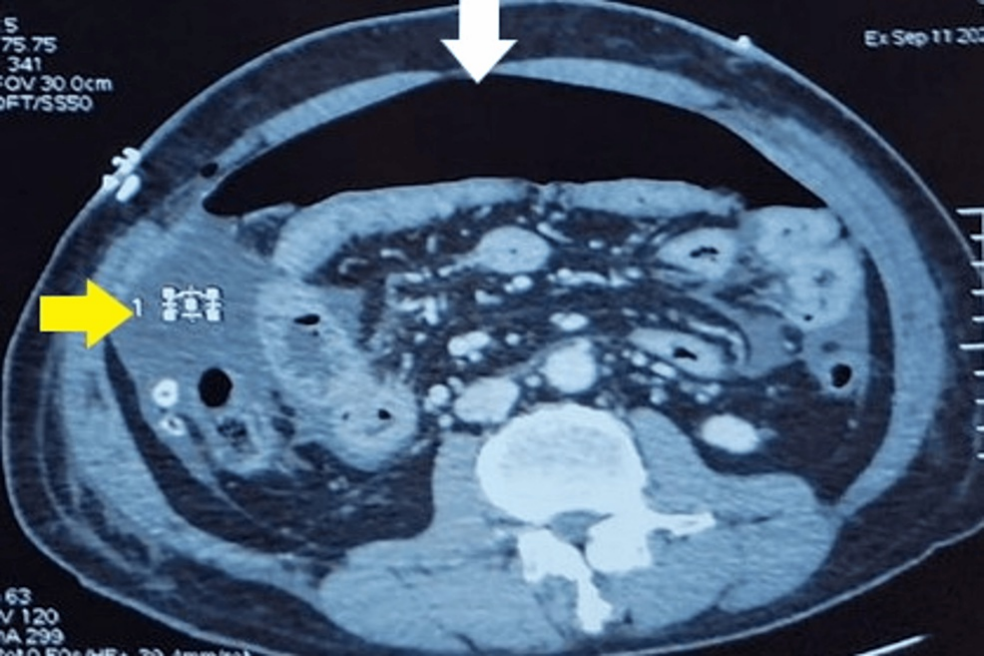
Computed tomography image showing free abdominal air (white arrow) and abdominal fluid (yellow arrow).

A 56-year-old male patient with a history of multifocal liver cancer (hepatocellular carcinoma) detected in April 2018 underwent three rounds of transarterial chemoembolization and one radiofrequency ablation and was deemed suitable for liver transplantation. The patient underwent an actual whole liver transplant from a brain-dead donor with blood type A. Following the transplant, the patient received intensive care treatment for three days. The posttransplant treatment regimen included prednisolone, Prograf 3 mg/3 mg, and Cellcept 750 mg/750 mg. The FK concentrations were maintained at 8-11 ng/mL. Pathology of the liver tumor revealed multifocal hepatocellular carcinoma, staged as pT2N0, with liver cirrhosis (F4) and chronic inflammation. The patient resumed light eating, but on the sixth-day posttransplant, he experienced diffuse abdominal pain, abdominal distension, and tenderness in the left half of his abdomen. An abdominal multislice CT scan revealed a hollow organ perforation with free gas (Figure 3).

**Figure 4:**
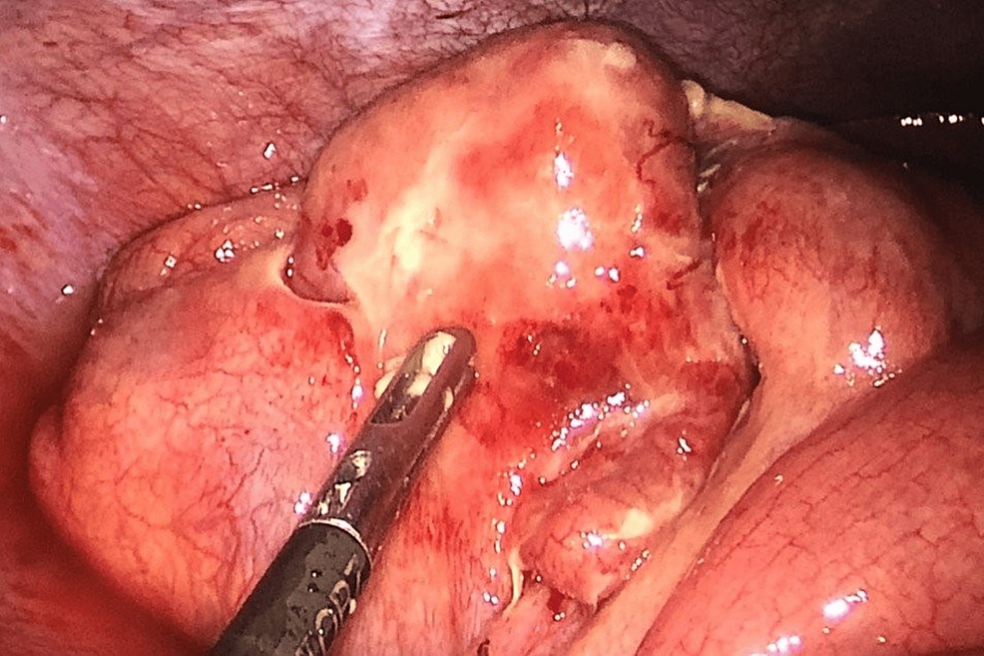
Image captured during laparoscopic surgery showing the perforation site in the sigmoid colon and many pseudomembranes attached to the intestinal wall.

Laboratory tests revealed a hemoglobin level of 84 g/L, white blood cell count of 15 G/L with 76% neutrophils, platelet count of 90 G/L, elevated liver enzymes (serum glutamic-oxaloacetic transaminase/serum glutamic-pyruvic transaminase: 207.62/581.34 IU/L), direct bilirubin of 75 µmol/L, blood creatinine of 150 µmol/L, and CRP of 120 ng/L. The patient underwent emergency surgery, which revealed intra-abdominal digestive fluid, concentrated pus in the left pelvic cavity and Douglas pouch, and minimal fluid below the liver (Figure 4).

**Figure 5:**
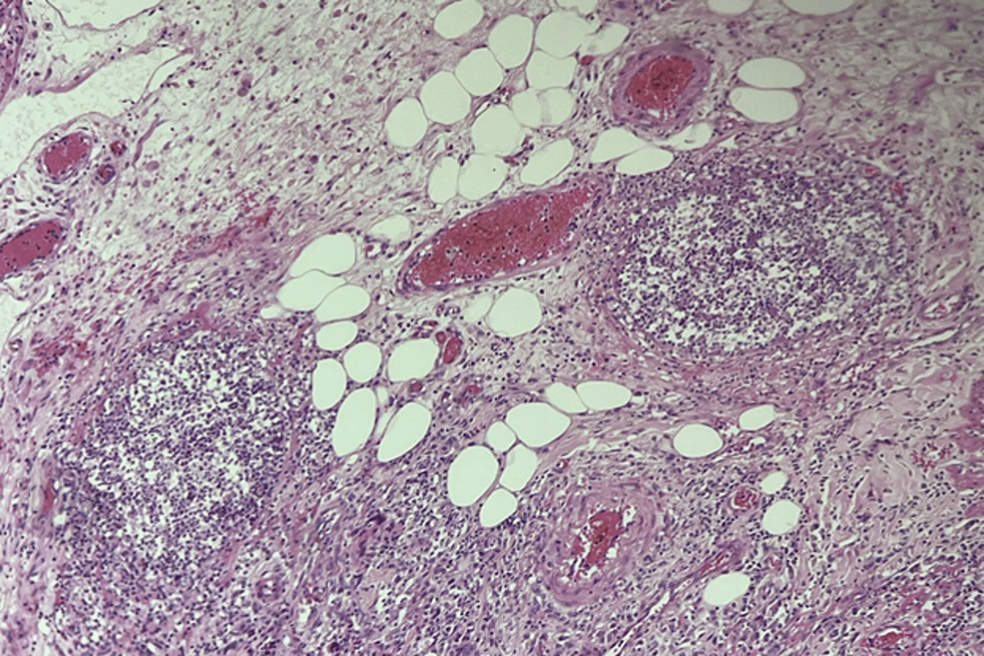
Image showing anatomic pathology with a purulent inflammatory lesion.

The cause was determined to be a sigmoid colon perforation, with a diameter of 1.5 cm and soft margins. A biopsy of the perforation edge revealed necrotic tissue with inflammatory exudate and some undetermined fibrous elements (Figure 5).

The mucosal glands appeared benign. The patient underwent two-layered closure of the perforation site, with the closure site near the abdominal incision line, leaving a 2 cm gap from the muscle layer to allow drainage, with sutures removed and retied once the perforation stabilized. The patient received intensive care treatment, a transfusion of two units of blood and two pools of platelets, a maintenance dose of Prograf 3 mg/3 mg, and a reduced dose of Cellcept to 250 mg/250 mg. Anatomic pathology revealed a purulent inflammatory lesion. The patient’s surgical course was stable, and he was discharged after 30 days.

Case 3: colonic perforation following kidney transplantation

**Figure 6:**
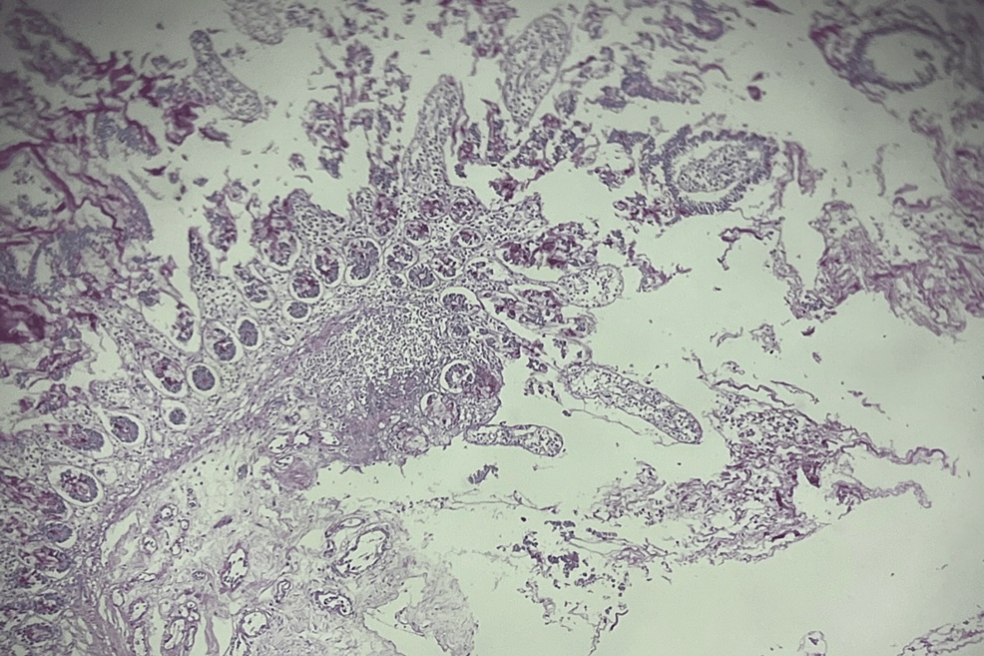
Biopsy results indicating ulceration of the thin ileal wall, pure atrophy, surrounding mucosa desquamation, poor fibrosis at the edge and base of the ulcer, and mucosal infiltration with an inflammatory exudate.

A 46-year-old female patient with end-stage renal failure due to chronic glomerulonephritis underwent right kidney transplantation via one artery and one vein from a living donor on May 10, 2017. In Vietnam, the resistance to cephalosporin antibiotics is about 70-80%. Therefore, when no antibiotic test is performed, meronem is used at a dose of 1 g (three vials) with slow intravenous infusion every six to eight hours/vial. The human leukocyte antigen match was 3/6. The patient was discharged after 16 days. The immunosuppressive triple-drug regimen included prednisone 20 mg, Prograf 3.5 mg/3.5 mg, and Cellcept 750 mg/750 mg, and FK levels were maintained at 11-12 ng/mL. Five days after discharge, the patient presented with nausea and abdominal pain around the ribs, leading to admission to the nephrology dialysis department for three days due to gastrointestinal disorder posttransplantation. She received antibiotic treatment with 3 g/day of a third-generation cephalosporin and 0.5 g/day of ciprofloxacin, fluid infusion, and oral lactitol (two sachets/day). After three days, the diarrhea worsened, and her blood creatinine level increased to 250 µmol/L, with a white blood cell count of 20 G/L, 86% neutrophils, hemoglobin of 76 g/L, and CRP of 150 g/L. An abdominal CT scan revealed free gas in the abdominal cavity, and she was diagnosed with diffuse peritonitis due to hollow organ perforation. Emergency surgery revealed minimal turbid fluid in the abdominal cavity and pseudomembrane formation due to a 0.5 cm perforation in the ileum. Biopsy results indicated ulceration of the thin ileal wall, pure atrophy, surrounding mucosa desquamation, poor fibrosis at the edge and base of the ulcer, and mucosal infiltration with an inflammatory exudate. The typical images of perforated ulcerative inflammation suggested corticosteroid use as a cause (Figure 6). Gastrointestinal disorders are one of the common side effects of immunosuppressive drugs for MMF (Cellcept), so the symptoms are easily confused as diarrhea and abdominal pain, especially in the first three months when the patient had just started taking the drug but had not tolerated it.

The patient underwent an ileostomy and continued treatment with a triple-drug regimen (prednisone + tacrolimus + MMF), with a reduced dose of MMF to 500 mg/500 mg combined with the antibiotic meropenem 3 g/day + fosfomycin 1 g/day for 14 days. The gastric mucosa was protected with Nexium for seven consecutive days after surgery. The patient did not require dialysis blood filtration. After two weeks, the patient’s intestinal stoma returned to the original site, and she was discharged on the 40th day in stable condition, with no fever. Her blood creatinine decreased to 120 µmol/L, with FK maintained at 10 ng/mL.

## Discussion

In solid organ transplants, such as kidney, liver, and heart, the prolonged use of immunosuppressive drugs increases complications such as infections, cancers, and gastrointestinal sequelae, affecting the posttransplant prognosis [3]. Therefore, patients are often not diagnosed with hollow organ perforation in the early posttransplant stage. In addition, posttransplant infection remains a major challenge in terms of diagnosis and treatment because infection symptoms are masked by the use of immunosuppressive drugs. As a result, the diagnosis and treatment of pineal infections are delayed, and the spectrum of infection in kidney transplant patients is wider than in the general population. According to the literature, gastrointestinal perforation complications are less frequent than other gastrointestinal disorders, such as gastroduodenal ulceration, esophagitis, nonspecific enteritis, acute pancreatitis, hepatobiliary diseases, colonic complications, and gastrointestinal cancers [4]. de'Angelis et al. reported a 2.5% rate of emergency laparotomy among 71,671 patients, with a gastrointestinal perforation complication rate of 9.2% [2]. The perforation locations were the colon (58.4%), small intestine (33.8%), and stomach and duodenum (7.8%). Other studies reported colonic perforation rates ranging from 1% to 10%. Dehghani et al. [4] reported a rate of 6.9%, Shaked et al. [5] reported a rate of 9.8% after liver transplantation, Merell et al. [6] reported a rate of 1.1% after heart transplantation; and Squiers et al. [7] reported a rate of 1-5% after kidney transplantation.

The causes and mechanisms of perforation include various factors, predominantly the adverse effects of corticosteroids (often early onset), inhibiting wound healing by suppressing surface coating formation, reducing mucosal cell turnover, impairing fibroblast repair activity, directly affecting the intestine by thinning the mucosal layer, and promoting bacterial overgrowth, leading to subsequent perforation [8]. Other late-onset causes include the use of immunosuppressive drugs such as azathioprine and steroids in patients with diverticulitis or a history of gastroduodenal ulceration [9]. Another significant cause is colonic perforation in kidney transplant recipients due to polycystic kidney disease (accounting for 57% of colonic perforation cases post-kidney transplant) [9]. Other causes include lymphoproliferative disorders due to CMV or infectious complications (e.g., CMV, *Clostridium difficile*). In kidney transplant patients, common gastrointestinal complications occurring posttransplantation are acute pancreatitis and visceral perforation, the two most dangerous complications requiring early detection and intervention. According to Singh et al., gastrointestinal complications include colonic ulcers (3%), acute pancreatitis (2.2%), acute diarrhea (18%), and prolonged ileus (58%), with most cases occurring within the first year (66%) [10].

Colonic complications are also mentioned among the gastrointestinal complications in transplant patients [3]. Clinical manifestations often include abdominal pain, fever, gastrointestinal disorders, weight loss, and sepsis syndrome. Performing a colonoscopy for definitive diagnosis and obtaining biopsy specimens for etiological investigation, unprepared abdominal X-ray, and CT scans are crucial in diagnosing colonic perforation. Case 2 is a case in point. Almost all of our gastrointestinal perforation cases were diagnosed late due to their rarity and atypical clinical and laboratory presentations, with these conditions diagnosed only when there was mucosal inflammation. Regarding management, emergency laparotomy and open surgery are required when there are complications of gastrointestinal perforation. Techniques may vary depending on the extent of the injury and the surgeon’s evaluation. According to Shaked et al., commonly used methods include segmental resection with end-to-end anastomosis (23.2%), closure of duodenal and colonic ulcers (9.4%), and colonic segmental resection and restoration of gastrointestinal continuity (54.3%) [5]. Hartmann’s surgery is less commonly used due to the risk of infection from artificial anal openings in transplant patients. In the case of colonic perforation after heart transplantation, after a thorough abdominal lavage, we also opted for segmental resection with end-to-end anastomosis to avoid increasing the risk of infection when a segment of the colon protrudes through the skin in solid organ transplant patients with mucosal inflammation. Patients with colonic perforation must be hospitalized for a long time due to a high risk of graft loss and mortality. According to Shaked et al., the mortality rate was 50% for primary perforations and 78% for recurrent perforations [5]. Most deaths occur due to late diagnosis, mucosal inflammation, and septicemia. Lin et al. [11] described four patients (2.7%) who experienced colonic perforation 9-14 days post-liver transplantation. In these patients, the perforation hole was sutured, the affected bowel segment was resected, and colonic continuity was established. Two patients recovered, and two experienced a recurrence. There were no deaths during surgery.

## Conclusions

Intestinal perforation is a relatively rare complication. Early diagnosis is challenging due to nonspecific clinical symptoms and signs. Considering the possibility of intestinal perforation and obtaining early abdominal CT imaging can help prevent delayed diagnosis.
